# Adherence to oral amoxicillin dispersible tablets in children with community-acquired pneumonia enrolled in clinical trials in Malawi

**DOI:** 10.1186/s41479-021-00089-4

**Published:** 2021-06-25

**Authors:** Amy Sarah Ginsburg, Susanne May

**Affiliations:** 1 University of Washington, Seattle, WA USA; 2 University of Washington Clinical Trial Center, Building 29, Suite 250, 6200 NE 74th Street, Seattle, WA 98115 USA

**Keywords:** Adherence, Community-acquired pneumonia, Amoxicillin, Dispersible tablets, Clinical trial, Africa

Pneumonia remains the leading infectious cause of child mortality worldwide. Despite highly effective interventions to prevent and treat pediatric pneumonia, globally, it is estimated that only 60% of children under 5 years of age with symptoms of pneumonia are taken to a healthcare provider [1]. Healthcare provider adherence with pneumonia treatment guidelines is reported to be poor in many settings, preventing some children who do visit a healthcare setting from receiving appropriate antibiotics. Furthermore, among those who are prescribed antibiotics, patient adherence to the complete treatment course is also frequently reported to be poor. As part of our Innovative Treatments in Pneumonia (ITIP) project, we conducted a secondary analysis to assess factors associated with adherence to amoxicillin DT in the setting of clinical trials [2, 3].

The ITIP project was conducted at Kamuzu Central Hospital and Bwaila District Hospital in Lilongwe, Malawi between June 2016 and April 2019 and included two prospective, double-blind, randomized controlled clinical trials evaluating the duration of amoxicillin dispersible tablet (DT) treatment for non-severe community-acquired pneumonia among children 2–59 months of age. Children in the ITIP1 fast-breathing pneumonia trial were randomized to either placebo DT (intervention) or amoxicillin DT (control) administered twice daily for 3 days, and children in the ITIP2 chest-indrawing pneumonia trial were randomized to either 3 (intervention) or 5 (control) days of twice-daily amoxicillin DT, with those in the 3-day group receiving placebo for the last 2 days of study participation. Adherence was defined as the proportion of study drug doses taken by the enrolled child of those that were expected; children who were switched to a different course of antibiotics or otherwise experienced treatment failure were not expected to continue the original course of study drug, and thus doses after such events were not counted toward the adherence denominator. For example, if an ITIP1 study participant was switched to a different treatment course after dose 4 and ingested 3 out of 4 expected study drug doses, their adherence was 75%. Similarly, if an ITIP2 study participant was switched to a different treatment course after dose 6 and ingested 3 out of 6 expected study drug doses, their adherence was 50%.

Upon enrollment and randomization, the blinded study staff prepared and administered the first dose of study drug and instructed the caregiver how to administer the remaining doses using customized job aids and patient instructions with pictograms [4]. Children were kept under observation in the hospital (2–24 h in ITIP1 and 48 h in ITIP2) and assessed for treatment failure prior to discharge. Instructions on how to administer the study drug were repeated at each visit. Children were followed in the hospital, in the clinic, or at home if they missed their scheduled clinic visits for 14 days with follow-up visits at Days 2, 4, 6 (ITIP2 only), and 14. At each scheduled and unscheduled visit both in the hospital and post-discharge, in addition to assessing the child for treatment failure or clinical relapse, study drug adherence information was collected from the child’s caregiver, and drug dosing and administration reviewed. Missed doses resulted in caregiver counseling and re-education on study drug administration. At the outcome assessment visit for each study, study drug pill counts were conducted, and unused study drug was collected.

In a secondary analysis of data from these two trials, we found the average percent of adherence to all treatment arms to be very high (> 99%; Table 1). Only 14 (1.2%) and 44 (1.5%) children had any non-adherence reported in ITIP1 and ITIP2, respectively. Because adherence was so high, we were not able to identify any substantive differences in the level of adherence between any of the subgroups and did not perform any regression analysis. High levels of adherence seen in our clinical trials likely do not translate directly to real-world adherence, as participation in a clinical trial may often increase adherence. Caregivers volunteer to participate and adhere to study treatment with the understanding that they will be monitored and followed closely. Effort was made in our trials to encourage high study drug adherence including caregiver education with pictorial instructions on how to administer study drug, ongoing counseling, and reminders as well as regular pill checks. Administration based on caregiver reports may also overestimate adherence due to social desirability bias. Clinical trials typically involve more healthcare provider one-on-one time with caregivers, as well as phone check-ins and home visits; these results are unlikely to be replicated in real-world settings.

**Table 1:** Baseline and background characteristics and average percent adherence among enrolled children

	ITIP1	Average percent adherence^a^	ITIP2	Average percent adherence^a^
	*n* = 1123	99.5	*n* = 2980	99.7%
Age (months)				
2–11, n (%)	392 (34.9%)	99.5	1728 (58.0%)	99.6
12–35, n (%)	507 (45.1%)	99.5	1015 (34.1%)	99.8
36–59, n (%)	224 (19.9%)	99.4	237 (8.0%)	99.7
Mean (SD)	21.3 (15.0)		13.5 (12.2)	
Gender				
Male, n (%)	524 (46.7%)	99.5	1642 (55.1%)	99.7
Female, n (%)	599 (53.3%)	99.5	1338 (44.9%)	99.6
Mother’s highest level of education				
None, n (%)	22 (2.0%)	100	100 (3.4%)	99.4
Primary, n (%)	497 (44.3%)	99.4	1698 (57.0%)	99.6
Secondary, n (%)	568 (50.6%)	99.5	1143 (38.4%)	99.8
Tertiary, n (%)	36 (3.2%)	100	39 (1.3%)	99.5
Any other children aged 0–10 years in same household				
Yes	660 (58.8%)	99.3	1718 (57.7%)	99.7
No	463 (41.2%)	99.7	1262 (42.3%)	99.6
Ill for more than 24 h before entering study, n (%)				
Yes	1061 (94.5%)	99.6	2610 (87.6%)	99.7
No	62 (5.5%)	98.4	370 (12.4%)	99.8
Axillary temperature ≥ 38 °C, n (%)				
Yes	335 (29.8%)	99.8	920 (30.9%)	99.6
No	788 (70.2%)	99.4	2060 (69.1%)	99.7
Any concurrent diagnosis				
Yes	374 (33.3%)	99.1	969 (32.5%)	99.5
No	749 (66.7%)	99.7	2011 (67.5%)	99.8
Any concomitant medication				
Yes	744 (66.3%)	99.3	1935 (64.9%)	99.6
No	379 (33.7%)	99.8	1045 (35.1%)	99.8
Any concomitant antibiotic				
Yes	33 (2.9%)	100	66 (2.2%)	99.7
No	1090 (97.1%)	99.5	2914 (97.8%)	99.7
Malaria, n (%)				
Yes	141 (12.6%)	98.8	267 (9.0%)	99.4
No	982 (87.4%)	99.6	2713 (91.0%)	99.7
Moderate acute malnutrition, n (%)				
Yes	18 (1.6%)	100	91 (3.1%)	99.6
No	1105 (98.4%)	99.5	2889 (96.9%)	99.7
Diarrhea, n (%)				
Yes	104 (9.3%)	99.4	410 (13.8%)	99.1
No	1019 (90.7%)	99.5	2570 (86.2%)	99.8

*n* number, *SD* Standard deviation

^a^ Average adherence = Caregiver reported percent of study drug doses taken of the possible doses provided prior to treatment failure

In a sub-analysis of a prospective cohort study also conducted in Malawi, non-adherence to cotrimoxazole was not uncommon among children aged 2–59 months with fast-breathing pneumonia diagnosed by community health workers at rural village clinics [5]. Poor adherence to prescribed medications is a frequent concern in healthcare settings and can lead to treatment failure or relapse, longer recovery times, increased costs, and antimicrobial resistance. In pediatrics, adherence can be particularly challenging due to issues around formulation and palatability; infants and young children are unable to swallow solid capsules and tablets or may refuse based on taste [6]. The involvement of caregivers can add another dimension or barrier to successful treatment and desired outcomes. Caregivers may discontinue medications based on their perception of their child’s symptoms or misunderstand administration instructions. Hundreds of individual factors may affect patient adherence to treatment, including those related to socio-economic status, the healthcare team and system, the disease condition, the prescribed therapy, and the patient [7]. Non-adherence to short-term treatments for acute conditions, including antibiotics for bacterial diseases, may have serious implications such as decreased treatment effectiveness and increased medical expenses.

Use of the DT formulation may have contributed to the high rate of adherence in our trials. Estimates from a cluster-randomized study in Kenya found higher adherence in children who received amoxicillin DT than in those who received amoxicillin oral suspension (84% vs. 39%), with equivalence in clinical outcomes and acceptability of use [8]. Alternative formulation types may be viable methods for increasing medication adherence. DT formulations as those used in our trials combine many advantages of both solid and liquid formulations, and were developed to be easy and cost-effective to store, transport, and dispense while also being simple to prepare and administer [9]. DT formulations do not require refrigeration, have smaller volumes and weights than oral suspensions, and can be dissolved in small amounts of breast milk or water by the caregiver at the time of administration. Amoxicillin DT tablets are less than 50 cents per treatment course, which may result in both cost-savings, better treatment outcomes, and higher adherence [9]. The World Health Organization recommends oral amoxicillin DT as first-line treatment for non-severe pneumonia in children 2–59 months of age.

Qualitative pediatric pneumonia research conducted in Malawi found that caregivers showed confusion around dosing and treatment durations, and found it more difficult to be adherent when multiple drugs required different dosing schedules or preparations, such as splitting pills, diluting medication in water, or preparing food to eat with the medication [10]. This suggests that, despite the ease of preparation of DT alone, when combined with another medication with a different formulation and/or different dosing schedule, overall adherence may suffer.

While clinical trials evaluating antibiotic treatment may not be ideal to evaluate adherence reflective of real-world clinical and home settings given the increased follow-up and monitoring, they nevertheless may provide some useful information. Further studies evaluating adherence to DT antibiotic treatment for pediatric pneumonia in low-resource settings should be completed, integrating considerations and observations of healthcare administrators and providers, and caregivers. Additional work may be necessary to explore the barriers and facilitators to implementing solutions like easier-to-use DT formulations in these settings. Our finding of high adherence to DT antibiotics for pediatric pneumonia treatment in a clinical trial setting suggest that high adherence is possible, and addressing the gap between research and real-life implementation may be beneficial in successfully preventing deaths from pneumonia.

## Data Availability

Upon request, deidentified participant data that underlie the results reported in this correspondence will be made available to researchers who provide a methodologically sound proposal following publication of the planned primary and secondary analyses. Proposals should be directed to the corresponding author.

## Abbreviations

DT: Dispersible tablet
ITIP: Innovative Treatments in Pneumonia
